# Small noncoding RNA GcvB is a novel regulator of acid resistance in Escherichia coli

**DOI:** 10.1186/1471-2164-10-165

**Published:** 2009-04-20

**Authors:** Ye Jin, Rory M Watt, Antoine Danchin, Jian-dong Huang

**Affiliations:** 1 Department of Biochemistry, Li Ka Shing Faculty of Medicine, University of Hong Kong, Pok Fu Lam, Hong Kong SAR, PR China; 2 Oral Biosciences, Faculty of Dentistry, University of Hong Kong, Pok Fu Lam, Hong Kong SAR, PR China; 3 Genetics of Bacterial Genomes, CNRS URA2171, Institut Pasteur, 28, rue du Docteur Roux, Paris Cedex 15, 75724, France

## Abstract

**Background:**

The low pH environment of the human stomach is lethal for most microorganisms; but not *Escherichia coli*, which can tolerate extreme acid stress. Acid resistance in *E. coli *is hierarchically controlled by numerous regulators among which are small noncoding RNAs (sncRNA).

**Results:**

In this study, we individually deleted seventy-nine sncRNA genes from the *E. coli *K12-MG1655 chromosome, and established a single-sncRNA gene knockout library. By systematically screening the sncRNA mutant library, we show that the sncRNA GcvB is a novel regulator of acid resistance in *E. coli*. We demonstrate that GcvB enhances the ability of *E. coli *to survive low pH by upregulating the levels of the alternate sigma factor RpoS.

**Conclusion:**

GcvB positively regulates acid resistance by affecting RpoS expression. These data advance our understanding of the sncRNA regulatory network involved in modulating acid resistance in *E. coli*.

## Background

The ability to survive the extremely low pH environment present within the stomach (pH values < 2.5) is essential for colonization of the intestine by commensals and for the pathogenesis of enteric bacteria. Three distinct acid resistance (AR) systems are responsible for protecting bacterial cells from acid shock [1-3]. AR system 1 is a glucose-repressed system and does not require any external amino acids to function at low pH [1]. Unidentified components of this system are controlled by the alternate sigma factor RpoS, with the underlying mechanism remaining unclear [1]. In contrast, AR systems 2 and 3 provide AR by consuming intracellular protons via the glutamate (AR2) and arginine (AR3) decarboxylation reactions [2,3]. AR2 is the most efficient AR system. It involves two isozymes of glutamate decarboxylase, encoded by genes *gadA *and *gadB*, and a membrane-associated antiporter (GadC) that exchanges external glutamate for the intracellular decarboxylation product γ-aminobutyric acid (GABA) [2].

AR2 is hierarchically regulated by multiple regulators, which include small noncoding RNAs (sncRNA) DsrA and possibly GadY. DsrA activates the expression of *rpoS *[4] and several AR genes including *hdeAB*, *gadAX*, and *gadBC *[5]. GadY, whose effects on AR have yet to be determined, controls the synthesis of GadX and GadW, which are regulators of the GadA and GadB glutamate decarboxylases [6,7]. In recent years, an increasing number of sncRNAs have been identified, but the functions of the vast majority of these are unknown. This prompted us to investigate whether sncRNAs in addition to DsrA and GadY, were involved in AR; and if so, to establish how they exerted their regulatory actions.

In the present study, seventy-nine snRNAs identified prior to 2006 [8-10] were selected for the construction of a single-gene knockout library within *E. coli *K12-MG1655. Comparison of each mutant with the wild type strain enabled us to identify the sncRNA GcvB as a previously unknown regulator of AR in *E. coli*. We demonstrate that GcvB positively regulates AR predominantly by upregulating the levels of the RpoS transcription factor (Sigma S). Our findings provide further insight into the regulatory roles played by sncRNAs in AR, which contributes to virulence of *E. coli*.

## Results

### Construction of single-sncRNA gene knockout mutant collection in *E. coli *K-12 MG1655

To systematically characterize sncRNAs, we established a single-sncRNA gene knockout library within *E. coli *K12-MG1655. Recombineering techniques were used to replace the target gene with a selectable chloramphenicol-resistance cassette, which was generated by polymerase chain reaction (PCR) using primers containing homology (45 nt) to the gene-flanking regions. This homologous recombination-based approach enabled precise gene deletion with minimal interference to adjacent genes (polar effects) [11,12]. In total, seventy-nine genes encoding sncRNAs identified prior to 2006 [8-10] were deleted individually from the MG1655 chromosome (see additional file 1).

### The sncRNA GcvB is a novel regulator of AR in *E. coli*

**Figure 1:**
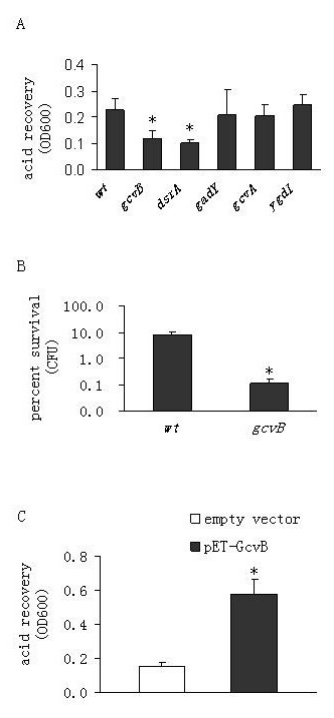
**Regulatory role of the small noncoding RNA GcvB in acid resistance in *Escherichia coli***. (*A*) MG1655-K12 wild type strain (wt) and various deletion mutants were grown in Luria Broth broth (pH 7.0) for 5 hr at 37°C. Cells were acid shocked at pH 2.0 for 30 min, and then neutralized by adding alkalinized LB (pH 9.3). Cells were allowed to grow for an additional 3 hrs before measurement of optical density at 600 nm (OD600). Two-tailed paired t tests were employed to compare each mutant with the wt in terms of acid resistance. (*B*) Because the *gcvB *mutant displayed a lower growth rate than the wt (data not shown), percent acid survival was determined to confirm the data of acid recovery measured as OD600. Cell viability was determined by plate counts and percent acid survival was calculated as the number of cfu remaining after the 30 min-acid treatment divided by the initial cfu at time zero (quoted as a percentage). (*C*) A plasmid expressing GcvB under the control of its native promoter was transformed into a *gcvB *mutant of *E. coli *MG1655. The mutant carrying an empty vector was used as a control. Cells were incubated for 5 hrs before being acid challenged for 15 min. Error bars represent standard deviation (* *P*-value < 0.05).

We investigated the involvement of sncRNAs in the AR of *E. coli *by challenging the sncRNA mutants as well as the wild type strain by incubation in LB medium at pH 2.0 (for 15 or 30 minutes), and comparing their percent survival and/or rates of recovery after pH neutralization (termed acid recovery). Unless otherwise stated, bacterial cells were grown for 5 hrs to the early stationary phase before the acid challenge. As expected, cells lacking DsrA, which is a known AR regulator, were sensitive to the acid challenge, confirming previous findings (Fig. 1A) [4,5]. However, deleting *gadY *did not affect acid survival (Fig. 1A), suggesting that GadY is not required for AR in spite of its positive control of GadX, a transcriptional activator of the GadA and GadB glutamate decarboxylases [6,7]. Interestingly, the absence of the sncRNA GcvB resulted in a significant reduction in acid survival (Fig. 1A, B). The same AR phenotypes in the sncRNA mutants were also observed during the exponential growth phase (data not shown), suggesting that GcvB exerts constant AR regulatory functions throughout the various growth phases. We also individually deleted *gcvA *and *ygdI *which are the two genes immediately adjacent to *gcvB*. Both mutants displayed comparable AR as the wild type (Fig. 1A). This shows that the observed acid vulnerability in the *gcvB *mutant is not due to the disruption of the activities of its adjacent genes.

Next, we cloned the gene encoding GcvB (together with its native promoter) into plasmid pET32a (+) and overexpressed the sncRNA in the *E. coli *MG1655 *gcvB *null mutant. The *gcvB *mutant carrying an empty vector served as a negative control. We found that overproducing GcvB restored the ability of the *gcvB *mutant to survive at low pH (Fig 1C). These observations together with the knockout data revealed that GcvB acts as a positive regulator of AR.

### The known GcvB target OppA plays a minor, if any, role in GcvB-mediated AR

**Figure 2:**
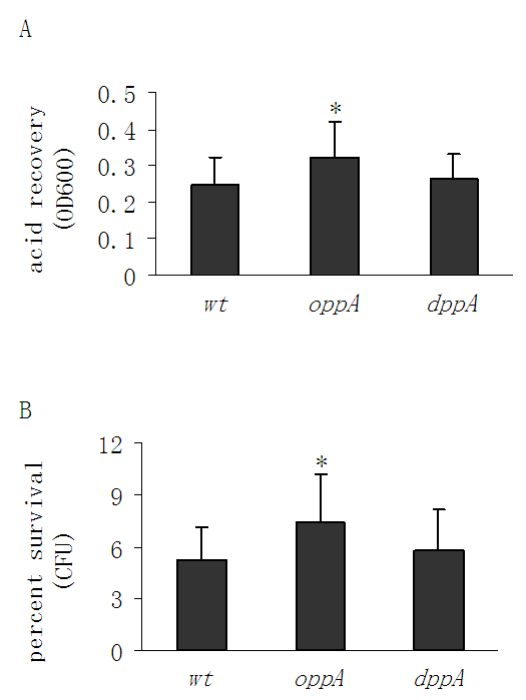
**Effects of individually deleting *oppA *and *dppA *on acid resistance in *Escherichia coli***. The acid-stress survival abilities of two individual deletion mutant strains lacking previously identified targets of GcvB were compared with those of the MG1655 wild type (wt) strain. The abilities of strains to survive a 30 minute acid shock were quantified by two methods: (*A*) acid recovery measured as optical density at 600 nm (OD600). (*B*) Percent survival determined by colony forming units (cfu). Error bars represent standard deviation (* *P*-value < 0.05).

GcvB has been demonstrated to repress the translation of *oppA *and *dppA *in *E. coli *by masking the ribosome binding sites of their mRNAs, which encode periplasmic substrate-binding proteins of ABC uptake systems for amino acids and peptides [13-15]. To determine whether these two proteins played a role in GcvB-mediated AR, we individually deleted their encoding genes, and examined the ability of the resultant mutants to survive at low pH. We found that deleting *oppA *slightly increased AR, as revealed by their acid recovery (OD600) and percent acid survival (cfu) levels, while the absence of *dppA *had little effect on AR (Fig. 2A, B). Thus DppA is not involved in GcvB-mediated AR, and OppA plays a minor, if any, role in this process. We reasoned that the GcvB sncRNA may be exerting its effects on AR via a previously unidentified molecular target.

### GcvB mediates AR by upregulating RpoS expression

**Figure 3:**
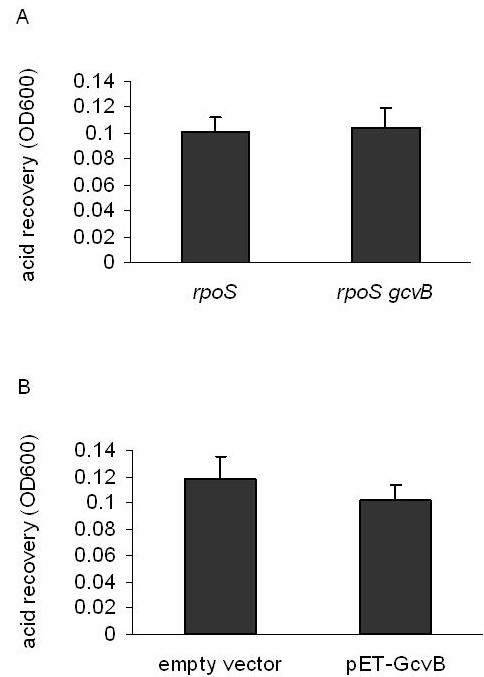
**Effects of deleting *rpoS *on GcvB-mediated acid resistance**. (*A*) Acid resistance in an *rpoS *mutant and an *rpoS gcvB *double mutant. (*B*) Effects of GcvB overproduction on acid resistance in an *rpoS *mutant. Cell cultures of *E. coli *MG1655 mutant strains were grown in LB broth (pH 7.0) at 37°C for 5 hrs and then acid challenged (pH 2.0) for 15 min. After neutralization by adding alkalinized LB (pH 9.3), cells were cultured for an additional 3 hrs before measurement of optical density at 600 nm (OD600 nm). Error bars represent standard deviation (* *P*-value < 0.05).

**Figure 4:**
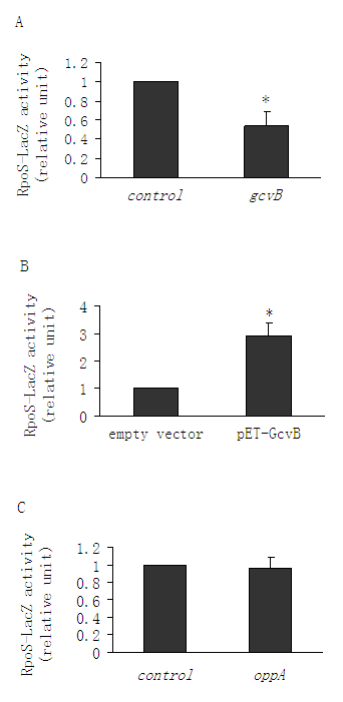
**Effects of GcvB sncRNA and OppA protein on the expression of RpoS in a chromosomal *rpoS-lacZ *reporter strain**. (*A*) Effects of deleting *gcvB *on the expression of an *rpoS-lacZ *reporter gene fusion in *E. coli *MG1655. (*B*) Effect of GcvB overproduction on the expression of the *rpoS-lacZ *fusion. GcvB production was driven by its native promoter on a pET32a plasmid construct. (*C*) Effect of deleting *oppA *on the expression of the *rpoS-lacZ *reporter. Beta-galactosidase activities due to translation of chromosomal *lacZ *fusions were normalized to those of MG1655 carrying an *rpoS-lacZ *fusion with or without an empty vector (controls). Error bars represent standard deviation (* *P*-value < 0.05).

RpoS is the central regulator of glutamate-dependent AR2 genes such as *gadA, gadBC *[1]*, gadX *[16]*, gadE *[17], and *gadY *[6,7]. It is also required for the AR1 system [1,18]. We therefore set out to determine whether GcvB regulated AR by modulating RpoS expression levels. We found that deleting *gcvB *did not lead to a further reduction in acid survival levels in an *rpoS *mutant background (Fig. 3A). More importantly, the absence of *rpoS *completely abolished AR elicited by the plasmid-encoded GcvB (Fig. 3B), suggesting that GcvB-mediated AR is dependent on RpoS. Next, an *rpoS-lacZ *translational fusion was constructed to examine whether the presence or absence of the GcvB sncRNA significantly affected *rpoS *expression levels. Deleting *gcvB *reduced the expression levels of the *rpoS-lacZ *fusion by 50% (Fig. 4A), whereas *gcvB *overexpression increased it 3-fold (Fig. 4B). Furthermore, deleting *oppA*, whose absence had previously been shown to slightly increase AR levels, had no effect on *rpoS-lacZ *expression (Fig. 4C). This indicates that the GcvB-mediated regulation of RpoS expression does not involve OppA.

**Figure 5:**
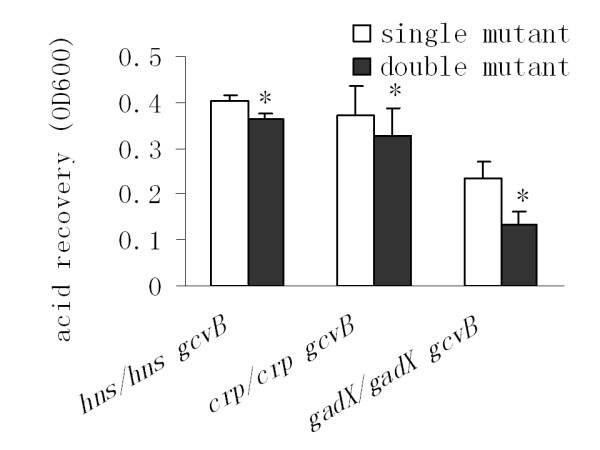
**Effects of deleting *gcvB *on acid resistance in mutants lacking *hns*, *crp *or *gadX***. Cell cultures of *E. coli *MG1655 mutant strains containing single deletions of the *hns*, *crp *or *gadX *genes were grown in LB broth (pH 7.0) at 37°C for 5 hrs and then acid challenged (pH 2.0) for 15 min (*gadX *mutant) or 30 min (*hns *and *crp *mutants). After neutralization by adding alkalinized LB (pH 9.3), cells were cultured for an additional 3 hrs before measurement of optical density at 600 nm (OD600 nm). Error bars represent standard deviation (* *P*-value < 0.05).

**Figure 6:**
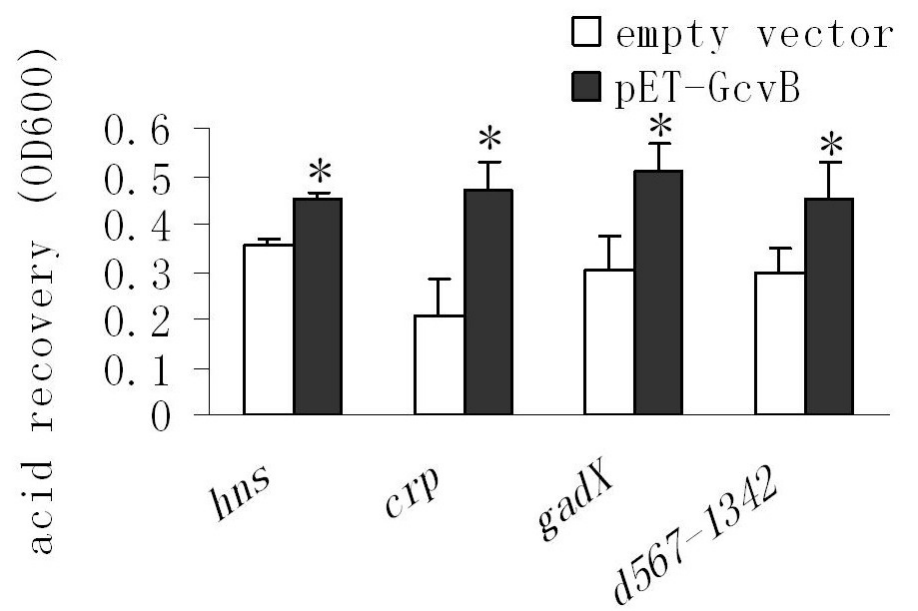
**Effects of GcvB overexpression on acid resistance in mutants lacking *hns, crp, gadX *or native promoters of *rpoS***. The mutant lacking the native promoters of *rpoS *was named d_567–1342. _Cell cultures of *E. coli *MG1655 strains containing single deletions were grown in LB broth (pH 7.0) at 37°C for 5 hrs and then acid challenged (pH 2.0) for 15 min (*gadX *mutant) or 30 min (*hns *and *crp *mutants and d_567–1342_). After neutralization by alkalinized LB (pH 9.3), cells were cultured for an additional 3 hrs before measurement of optical density at 600 nm (OD600 nm). Error bars represent standard deviation (* *P*-value < 0.05).

We then investigated whether GcvB indirectly controls RpoS production via an upstream regulator. In the literature, there are numerous reports outlining mechanisms by which the synthesis of RpoS is regulated at the transcriptional [19-22], translational and posttranslational levels [23-25]. Of these regulators of RpoS expression, Crp [20], H-NS [23], GadX [22], and a hybrid sensor kinase called BarA [21] have previously been associated with AR. Although BarA acts as a positive regulator of *rpoS *transcription, its effect is restricted to the exponential phase. It is therefore unlikely that BarA is a potential target for GcvB. Consequently, we examined whether GcvB exerts its positive control of RpoS through Crp, H-NS or GadX. We found that deleting *gcvB *reduced acid survival levels in an *hns *mutant background. This was also the case for *crp*- or *gadX*-deficient strains (Fig. 5). Consistent with the knockout data, *gcvB *overexpression increased AR levels in the *hns*-, *crp*- or *gadX*-deficient strains (Fig. 6). These results suggest that Hns, Crp and GadX are not involved in the GcvB-mediated regulation of *rpoS *expression.

*rpoS *transcription is driven by a major promoter *rpoSp *and two weak promoters *nlpDp1 *and *2 *[26]. To examine whether GcvB affected *rpoS *expression at the transcriptional level, we deleted the region spanning the *rpoSp*, *nlpDp1 *and *2 *promoter sites (Fig. 4). As removing these promoters will eliminate *rpoS *expression, a constitutive promoter encoded in the knockout cassette (described in Materials and Methods) was used to drive *rpoS *transcription in the absence of *rpoSp*, *nlpDp1 *and *2*. The resulting mutant was referred to as d_567–1342_. As shown in Fig. 6, replacing the native promoters of *rpoS *with this heterologous promoter had little effect on GcvB-induced AR. These results indicate that GcvB does not regulate *rpoS *expression at the transcriptional level.

## Discussion

In this study, we constructed a library comprising single-gene knockout mutants of seventy-nine sncRNA genes in *E. coli*. We used this mutant collection to identify sncRNAs involved in acid resistance (AR) within this bacterium. Consequently, we were able to identify the sncRNA GcvB as a novel regulator of AR.

DppA and OppA, which were previously identified as targets of GcvB, were shown not to be responsible for GcvB-mediated AR. While the *dppA *deletion did not affect AR, the absence of OppA slightly improved the ability of *E. coli *to survive under extreme acid conditions. Since GcvB-mediated inhibition of *oppA *and *dppA *only weakly increased AR, we reasoned that the GcvB sncRNA may be regulating AR via some other mechanistic pathway.

Upon *rpoS *deletion, GcvB had no influence on AR. Furthermore, we found that GcvB positively regulated *rpoS *expression. Deleting *oppA *had no effect on the levels of RpoS, providing evidence that the GcvB-mediated control of RpoS does not involve the OppA protein. Also, GcvB does not regulate RpoS via H-NS, Crp, or GadX; as GcvB-mediated AR was unaffected when these genes were singularly deleted. The region spanning from -567 to -1342 nt harbors all three of the of the *rpoS *promoters (*rpoSp*, *nlpDp1 *and *2*). Replacing these native promoters with the constitutive promoter from a Cm knockout cassette had no affect on GcvB-mediated AR. This suggests that GcvB does not regulate *rpoS *expression at the transcriptional level.

Like GcvB, the sncRNAs DsrA and RprA also act as positive regulators of AR in *E. coli*. DsrA and RprA bind to the same region of the 5' leader of *rpoS *mRNA. A sequence within this region was previously predicted to form a self-inhibitory stem loop structure that probably occludes the Shine-Dalgarno ribosome binding site and prevents translation [24,27]. DsrA [24,27] and RprA [28,29] bind to this stem-loop structure, free the ribosome binding site, and thereby mediate *rpoS *translation. This region also contains RNase III cleavage sites that result in rapid decay of the *rpoS *transcript [30]. The base-pairing of DsrA within this region stabilizes *rpoS *mRNA by creating an alternative RNase III cleavage site [30]. As for GcvB, it regulates *rpoS *expression by an unknown mechanism. Computational sequence analyses using the RNAhybrid server [31] have indicated that GcvB does not contain any extensive regions of sequence complementary to the 5' leader of *rpoS *mRNA (data not shown). As such, the mechanism by which GcvB regulates *rpoS *expression remains a subject of further investigation.

## Conclusion

Here we reveal that the sncRNA GcvB positively regulates the ability of *E. coli *to survive low pH conditions by upregulating the expression of RpoS. Our findings provide insight into the control of AR by GcvB. However, further detailed studies will be required to investigate the precise nature of the GcvB/*rpoS *interaction at the molecular level.

## Methods

### Bacterial strains, plasmids, and growth conditions

*E. coli *K12-MG1655 was used for all gene deletion and overexpression experiments. The *E. coli *expression vector pET32a (+) (Novagen, Madison, WI) was used for the overexpression of sncRNAs. All bacterial strains were grown at 37°C, with shaking at 230 rpm, in Luria-Bertani (LB) medium supplemented with antibiotics when required. Bacterial growth was monitored by measuring optical density at 600 nm (OD600). The antibiotics ampicillin (50 ug/ml), kanamycin (50 ug/ml), and chloramphenicol (12.5 ug/ml) were used for selection.

### Gene deletions

Gene deletion mutations were constructed using the λ-Red recombination system. *E. coli *MG1655 was transformed with plasmid pSim6 (a gift from Dr. Donald Court) [32] on which the expression of the λ recombination proteins is induced at 42°C. PCR fragments encompassing a loxP-cm-loxP ('floxed') chloramphenicol resistance cassette with homology (45 nt) to the regions immediately flanking each deletion locus were transformed into MG1655 harboring pSim6. After induction of λ*red*, recombinants were selected for chloramphenicol (cm) resistance, and were further verified by colony PCR.

### Gene cloning

The selectable loxP-cm-loxP cassette was first inserted immediately after the stop codon of the gene of interest on the chromosome (as described above). We then used recombineering to 'clone' this gene of interest preceded by its native promoter and 'floxed' cm cassette downstream of the T7 promoter in a pET32a expression vector. Specifically, we PCR amplified the gene of interest (including its native promoter) and adjacent 'floxed' cm cassette using primers that contained homology (45 nt) to the plasmid insertion site. The PCR product and expression vector were co-transformed into DY330[12] after induction of λ*red*. Recombinants were selected for chloramphenicol resistance and verified by PCR and sequencing. The native promoter drove the gene overexpression in MG1655 without induction.

### Construction of chromosomal *lacZ* translational fusions and beta-galactosidase assays

First, the loxP-cm-loxP selectable cassette was inserted immediately after the stop codon of the *lacZ *gene on the MG1655 chromosome using λ*red *recombination (as described above). Next, the lacZ-loxP-cm-loxP cassette was PCR amplified and inserted (in frame) immediately prior to the stop codon of the target gene on the chromosome. The inserted *lacZ *fragment started from the 8^th ^codon of *lacZ *gene and co-transcribed and -translated with the fused genes. The expression of gene*-lacZ *fusions was quantified using a beta-galactosidase assay kit from Thermo Fisher Scientific (Rockford, Illinois, USA).

### Acid resistance assay

Overnight cultures were diluted 1:50 and incubated at 37°C for 5 hrs in LB medium. 3 volumes of acidified LB medium (pH 1.9) was then added to 1 volume of the cell culture so that the final pH value was approximately 2.0. The acid challenge was maintained for 15 min for cells that were acid sensitive, and 30 min for acid-resistant cells. The acid challenge was stopped by adding 3 volumes of alkalinized LB medium (pH9.3), making the final pH value approximately 7.0. For samples that displayed similar growth rates, the neutralized cell cultures were allowed to grow for 3 hrs following the acid challenge, and acid survival levels were determined by measurement of optical density at 600 nm (OD600, termed acid recovery). When samples with different growth rates were examined, cell viability was determined by plate counts and percentage acid survival was calculated as the number of number of colony forming units (cfu) remaining after the acid treatment divided by the initial cfu at time zero.

### Statistical analysis

Two-tailed paired t-tests were used for the comparison of means obtained from the beta-galactosidase and acid survival assays. *P *values of < 0.05 were considered statistically significant.

## Abbreviations

AR: acid resistance; sncRNA: small noncoding RNA; UTR: unstranslated region.

## Authors' contributions

YJ planned and designed the experiments, performed the experiments and the data analysis, wrote the main draft of the paper, and generated the figures. RW conceived of the study and helped to draft the manuscript. AD aided in the experimental design and in editing the manuscript. JH provided guidance over the entire project, and aided in data analysis and editing of the manuscript. All authors read and approved the final manuscript.

## Supplementary Material

Additional file 1 **Small noncoding RNAs (sncRNA) that were selected for construction of a single-sncRNA gene knockout library within *E. coli *K12-MG1655**. This table summarizes positions and functions of the small noncoding RNAs that were included in a single-sncRNA gene knockout library within *E. coli *K12-MG1655. Click here for file
