# Supplementary material for: Small noncoding RNA GcvB is a novel regulator of acid resistance in Escherichia coli
Source: BMC Genomics. 2009 Apr 20;10:165. doi: 10.1186/1471-2164-10-165 (PMC2676305; doi:10.1186/1471-2164-10-165)
Supplement: Additional file 1 — Small noncoding RNAs (sncRNA) that were selected for construction of a single-sncRNA gene knockout library within E. coli K12-MG1655. This table summarizes positions and functions of the small noncoding RNAs that were included in a single-sncRNA gene knockout library within E. coli K12-MG1655. [file 1471-2164-10-165-S1.doc]

Additional file 1. Small noncoding RNAs that were selected for construction of a single-sncRNA gene knockout library within *E. coli* K12-MG1655.

| **sncRNAs** | **map position** | **functions** |
| --- | --- | --- |
| 4.5S (ffs) | 475672 -> 475785 | formation of the signal recognition particle |
| 6S RNA (SsrS) | 3054005 -> 3054187 | repression of expression from sigma70- and sigmaS-dependent promoters. |
| CrpT | 3483841 <- 3484141 | regulation of *crp* |
| CsrB | 2922178 <- 2922537 | inhibition of the activity of CsrA |
| CsrC | 4049059 -> 4049303 | inhibition of the activity of CsrA |
| DicF | 1647406 -> 1647458 | inhibition of FtsZ mRNA translation |
| DsrA | 2023251 <- 2023337 | activation of *rpoS* and inhibition of H-NS translation. |
| FimA 3' | 4541245 -> 4541276 | unknown |
| GadY (IS183) | 3662887 -> 3662991 | positive regulation of *gadX* |
| GcvB | 2940718 -> 2940922 | repression of *oppA* and *dppA* translation. |
| GlnA 3' | 4054150 <- 4054201 | unknown |
| IS061 | 1403676 <- 1403833 | unknown |
| IS092 | 1985863 <- 1986022 | unknown |
| IS102 | 2069339 -> 2069542 | unknown |
| IstR-1 | 3851141 <- 3851215 | repression of *tisB* translation |
| IstR-2 | 3851141 <- 3851280 | repression of *tisB* translation |
| LysC 5' | 4231081 <- 4231116 | unknown |
| MgtA 5' | 4465125 -> 4465161 | unknown |
| MicA (SraD) | 2812823 -> 2812897 | repression of *ompA* expression |
| MicC | 1435145 -> 1435252 | repression of *ompC* expression |
| MicF | 2311106 -> 2311198 | negative control of *ompF* translation and *ompF* mRNA stability |
| OmrA | 2974124 <- 2974211 | unknown |
| OmrB | 2974332 <- 2974407 | unknown |
| OxyS | 4156308 <- 4156417 | regulation of *fhlA* and *rpoS* |
| QUAD1d (tp8) | 3192773 <- 3192922 | unknown |
| QUAD1e (rygE) | 3193150 <- 3193262 | unknown |
| RdlA | 1268546 -> 1268612 | an antisense regulatory RNA part of a toxin-antitoxin pair |
| RdlB | 1269081 -> 1269146 | an antisense regulatory RNA part of a toxin-antitoxin pair |
| RdlC | 1269616 -> 1269683 | an antisense regulatory RNA part of a toxin-antitoxin pair |
| RdlD | 3698158 -> 3698221 | regulation of *ldrD* translation. |
| RnpB | 3268238 <- 3268614 | essential for viability |
| RprA | 1768396 -> 1768500 | required for RpoS production in response to osmotic shock |
| Rpsp 5' | 2744224 <-2744254 | unknown |
| RybA | 852175 <- 852263 | unknown |
| RybB | 887199 <- 887277 | repression of *rpoS* expression in minimal media |
| RybD | 764272 -> 764376 | unknown |
| RydB | 1762737 <- 1762804 | repression of *rpoS* expression in minimal media |
| RydC | 1489456 <- 1489701 | degradation of *yejABEF* mRNA. |
| RyeA (SraC) | 1921090 -> 1921338 | unknown |
| RyeB | 1921188 <- 1921308 | unknown |
| RyeC | 2151299 -> 2151447 | unknown |
| RyeD | 2151634 -> 2151776 | unknown |
| RyeE | 2165136 -> 2165221 | repression of *rpoS* expression in LB |
| RyeF | 1956156 <- 1956544 | unknown |
| RyfA (PAIR3) | 2651877 -> 2652180 | unknown |
| RyfB | 2698079 <- 2698397 | unknown |
| RyfC | 2698540 -> 2698616 | unknown |
| RyfD | 2732193 <- 2732323 | unknown |
| RygC | 3054837 -> 3054987 | unknown |
| RyhB (SraI) | 3578946 <- 3579039 | regulation of acnA, fumA, ftnA, bfr, and sodB; repression of rpoS in minimal media |
| RyiA (SraJ) | 3984455 -> 3984626 | unknown |
| RyjA (SraL) | 4275950 <- 4276089 | unknown |
| RyjB | 4525545 -> 4525634 | unknown |
| RyjC (SymR) | 4577858 -> 4577934 | repression *symE t*ranslation |
| SgrS | 77367 -> 77593 | destabilization of *ptsG* mRNA. |
| SokB | 1490143 -> 1490195 | regulation of the translation of MokB and HokB. |
| SokC | 16952 -> 17006 | regulation of the translation of MokB and HokB. |
| SokE | 606957 <- 607015 | unknown |
| SokX | 2885376 -> 2885431 | unknown |
| Spf (Spot42) | 4047922 -> 4048030 | repression of *galK* translation |
| Spy 3' | 1823084 <- 1823131 | unknown |
| SraA | 457925 <- 458111 | unknown |
| SraB | 1145812 -> 1145980 | unknown |
| SraG | 3309247 -> 3309420 | unknown |
| SraH (RyhA) | 3348599 -> 3348706 | repression of *rpoS* expression in minimal media |
| SroA | 75516 <- 75608 | unknown |
| SroB/RybC | 506428 -> 506511 | unknown |
| SroC | 685904 <- 686066 | [unknown](../../../../C:%5CDocuments%20and%20Settings%5CJin%20Ye%5CDesktop%5Cpaper%20components%5C4th%20version%5Csupplementary%20table%201S.xls#RANGE!14602901%23RANGE!14602901) |
| SroD | 1886041 <- 1886126 | unknown |
| SroE | 2638617 <- 2638708 | unknown |
| SroF (tke1) | 2689214 <- 2689362 | unknown |
| SroG | 3182592 <- 3182740 | unknown |
| SroH | 4188350 <- 4188510 | unknown |
| SsrA (Tm RNA) | 2753615 -> 2753977 | An ssrA mutant exhibits a growth defect and a motility defect. |
| T44 | 189712 -> 189847 | unknown |
| Tp2 | 122857 <- 123016 | unknown |
| Tpke11 | 14080 -> 14168 | unknown |
| Tpke70 | 2494216 <- 2494651 | unknown |
| ZipA 5' | 2529267 <- 2529300 | unknown |
